# Tailored Meal-Type Food Provision for Diabetes Patients Can Improve Routine Blood Glucose Management in Patients with Type 2 Diabetes: A Crossover Study

**DOI:** 10.3390/nu16081190

**Published:** 2024-04-17

**Authors:** Dong Hoon Jung, Jae Won Han, Hyeri Shin, Hee-Sook Lim

**Affiliations:** Department of Gerontology, Age Tech-Service Convergence Major, Graduate School of East-West Medical Science, Kyung Hee University, Yongin 17104, Republic of Korea; jdh1106@khu.ac.kr (D.H.J.); ltc.shinhyeri@gmail.com (H.S.)

**Keywords:** diabetes mellitus, type 2, diet therapy, blood glucose self-monitoring, health care

## Abstract

This study aimed to determine whether patients with type 2 diabetes can benefit from a meal plan designed based on diabetes management guidelines to improve blood glucose levels. Participants were divided into intervention and control groups. The intervention group consumed a diabetic diet for 2 weeks, while the control group consumed their normal diet. After 2 weeks, the groups switched their dietary regimens. The participants’ demographic and clinical characteristics were evaluated, including factors such as blood pressure, blood lipid levels, weight and waist circumference, blood glucose levels (self-monitored and continuously monitored), nutritional status, and blood-based markers of nutrient intake. The dietary intervention group improved waist circumference, body fat percentage, low-density lipoprotein cholesterol, triglyceride levels, and glucose. The energy composition ratio of carbohydrates and proteins changed favorably, and sugar intake decreased. In addition, the proportion of continuous glucose monitoring readings within the range of 180–250 mg/dL was relatively lower in the intervention group than that of the control group. Meals designed based on diabetes management guidelines can improve clinical factors, including stable blood glucose levels in daily life, significantly decrease the carbohydrate energy ratio, and increase the protein energy ratio. This study can help determine the role of dietary interventions in diabetes management and outcomes.

## 1. Introduction

Diabetes stands as a significant worldwide public health issue [1]. The International Diabetes Federation’s data from 2021 highlights that around 537 million adults across the globe were living with diabetes, representing close to 10% of the adult population internationally [2]. These figures are on an upward trajectory, with expectations set for them to rise to 643 million by the year 2030 and further to 783 million by the year 2045 [2]. In the context of South Korea, the Korean Diabetes Association reported that, as of the year 2020, the incidence of diabetes among the adult population aged 30 years and above was 16.7%. This prevalence increases significantly in the older population, with adults aged 60 years and above experiencing a diabetes prevalence rate of 30.1% [3]. In terms of prediabetes, the rates were considerably high as well, recorded at 44.3% for adults aged ≥30 years and even higher at 50.4% for those in the ≥60 years age bracket [3]. Despite these high prevalence rates, the treatment uptake was not as high as it should be, with only 60% of those in need of medical intervention receiving it [3]. Particularly concerning is the diabetes management rate among the elderly, individuals aged 65 years and above, which was only 28.3% [3], highlighting a critical area for healthcare improvement and intervention.

Diabetes is a chronic condition necessitating daily management [4], including self-monitoring of blood glucose levels, which can be supported by the use of continuous glucose monitoring (CGM) devices [4,5]. These devices are associated with decreased risks of both hypoglycemia and hyperglycemia [6]. CGM technology involves a sensor placed under the skin, which measures glucose levels in the interstitial fluid at regular intervals ranging from 1 to 5 min [6]. This medical device serves as a complement to self-monitoring of blood glucose and addresses the shortcomings associated with HbA1c measurements [6]. Several studies have reported that the employment of CGM devices leads to enhanced control over blood glucose levels and a reduction in the occurrence of hypoglycemic events [7,8].

For individuals living with diabetes, including those who are on medication, the meticulous planning of meals and the strategic management of their nutritional intake stand as pivotal elements in the regulation of blood glucose levels and the broader spectrum of health management [9]. Diet management affects short-term glycemic control and cardiovascular disease risk factors [4]. In the long term, an optimum diet can improve the quality of life and reduce healthcare costs [4,9]. Functional foods specifically designed for patients with diabetes have been developed both domestically and internationally. However, in this context, supplements alone are not sufficient, and patients require optimal meal plans to support their health effectively. In Korea, diets for diabetes management are regulated within the framework of a standard specification system for foods intended for medical purposes, specifically addressing the dietary needs of patients with diabetes [10].

Diabetes-specific diets that are tailored to individual needs can help improve blood glucose levels and other health indicators among the target population [11,12,13]. In cross-experimental studies using Home Meal Replacement (HMR)-type dietary formats for patients with diabetes, HMR diets positively affected weight, body mass index, waist circumference, and lipid profile [11]. In a study involving patients with type 1 diabetes, CGM monitoring of blood glucose level fluctuations during low-carbohydrate diet periods revealed lower blood glucose levels and improved CGM-based blood glucose indicators [12]. In addition, in a study involving the consumption of delivery-type diabetes meal boxes in Korea, significant improvements were observed during the intervention period in the average blood glucose levels and time spent within the target blood glucose range (70 mg/dL and 180 mg/dL). These findings highlight the positive impact of tailored diabetes diets on blood glucose management and overall health indicators [13].

Therefore, this study aimed to provide a set of diabetes-specific meals to older adults in South Korea, where the prevalence of diabetes is high. The aim of this study was to demonstrate the effectiveness of diabetes-specific meal plans and to monitor intervention improvement.

## 2. Materials and Methods

### 2.1. Study Design and Participants

**Figure 1:**
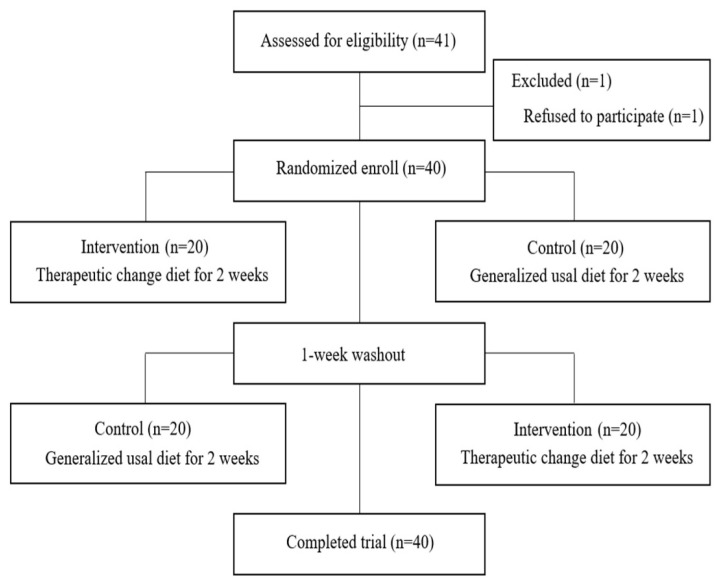
Study flowchart.

This study was a crossover trial. Participants aged 40–75 years with type 2 diabetes mellitus or prediabetes (impaired fasting glucose and impaired glucose tolerance) were recruited from local community health centers and hospitals. A total of 41 individuals were initially recruited; however, one person who declined to participate was excluded. Consequently, 40 participants were enrolled in this study. To ensure random group assignment, participants were allocated to either the intervention or control group using a randomization table based on sex, age, and duration of diabetes. At this stage, the groups were comparable in terms of sex, age, duration of diabetes, treatment, and educational level distribution. A flowchart of the study is shown in Figure 1.

Participants were selected based on their ability to consume regular meals at least twice a day [14], a physician-confirmed diagnosis, and ongoing medication or lifestyle therapy for diabetes. Individuals who had kidney disease as a complication of diabetes, had been hospitalized for high or low blood glucose, had a daily energy requirement exceeding 3000 kcal, had plans for hospitalization or surgery that could affect dietary intake, engaged in high-intensity regular exercise three or more times a week, or had a history of high-risk alcohol consumption (one-time alcohol intake of 60 g for men and 40 g for women) in the past 6 months were excluded. The process of selecting subjects who met the daily nutritional requirements was included, considering the energy provided per meal. The research period, including diet provision and data collection, was from September to November 2023. Here, 40 participants were divided into the intervention and control groups, with 20 participants in each group. The participants consumed their respective diets for 2 weeks each, followed by a 1-week washout period [15] before crossing over to consume the other diet. There were no dropouts, resulting in a final sample size of 40. The study aims were explained to the participants who consented to participate in the study. The study protocol was approved by the Ethics Committee of our institute (approval number: KHGIRB-23-383).

### 2.2. Diet Composition and Provision

Participants consumed three meals a day; however, in the intervention group, diabetes-specific food products were provided for two meals a day. The food products included 10 high-protein, low-sugar meal boxes (CJ CheilJedang Co., Seoul, Republic of Korea, Supplementary Table S1). These 10 types of meals are composed of an average of 573.5 kcal (range: 510 to 635 kcal), with an average serving amount of 30.4 g of protein and 73 g of carbohydrates per meal. They are complete meal boxes, including both main dishes and side dishes. Detailed information about each of these 10 food products is provided in Supplementary Table S1. These meals were provided in three separate deliveries to allow for two meals per day for two weeks. The remaining meals were consumed daily in the intervention group. The control group consumed three regular daily meals. To improve compliance and prevent errors during the research period, all participants received personalized education on diabetic dietary management from a clinical dietitian before the study began. Personalized nutritional requirements were calculated to raise awareness about appropriate daily and meal-specific intake amounts. The participants were instructed to avoid excessive consumption of fatty fast foods, alcohol, and frequent consumption of sugary beverages. During the study period, dedicated investigators conducted phone monitoring to ensure that the delivered meals were properly consumed and to address any questions or concerns.

### 2.3. Participant Characteristics

Comprehensive data were gathered regarding the demographic and clinical attributes of the participants. This included a wide range of information such as gender, age, marital status, level of education, the average length of time since diabetes diagnosis, the methods of treatment currently being employed, and the presence of any additional health conditions or comorbidities. Moreover, lifestyle factors that could influence health outcomes, such as smoking habits, alcohol consumption levels, and the extent of physical activity or exercise, were also collected.

### 2.4. Outcome Assessment

The survey variables were broadly categorized into cardiovascular and nutritional risk factors. The measured cardiovascular risk factors were weight, waist circumference, body fat percentage, diastolic blood pressure, systolic blood pressure, total cholesterol, low-density lipoprotein (LDL) cholesterol, high-density lipoprotein (HDL) cholesterol, triglycerides, and self-monitoring of blood glucose (SMBG). Anthropometric measurements were performed using a tape measure, InBody S10 (InBody Co., Seoul, Republic of Korea), and a blood analyzer (Afinion2 analyzer, Abbott Co., Green Oaks, IL, USA). Lipid tests were performed using a blood analyzer (Afinion2 analyzer, Abbott Co., Green Oaks, IL, USA), and blood pressure was measured by taking readings at rest using a portable blood pressure monitor (Citizen CH-452, Citizen Co., Tokyo, Japan), and the average value was used. Self-monitoring blood glucose measurements were obtained with individual glucometers at least four times a day, including readings obtained 2 h after meals and before bedtime, and the analysis was based on the average recorded values. Nutritional factors were assessed using the Nutrition Quotient [16], a tool developed by the South Korean Ministry of Health and Welfare to evaluate adult dietary habits. This questionnaire includes 20 questions designed to determine a person’s diet and lifestyle patterns. The evaluation results are categorized into overall nutritional scores, balance, moderation, and practice.

### 2.5. Nutrient Intake

Throughout the research period, study participants documented every item of food and beverage they consumed, employing a dietary record method to ensure comprehensive tracking of their intake. This recorded data was then reviewed by trained dietitians. The daily nutrient intakes of each participant were determined with precision, utilizing specialized nutritional analysis software. (Can pro-6.0, Web ver.; Korean Nutrition Society, Seoul, Republic of Korea) for a period of 3 days (two weekdays and one weekend day).

### 2.6. Glycemic Control

This study involved both SMBG and CGM. CGM can capture blood glucose level fluctuations over 24 h, enabling patients to analyze and respond to them. In this study, CGM (FreeStyle Libre, Abbott Co., Green Oaks, IL, USA) devices and activity sensors were used, and participants were encouraged to maintain consistency in activity levels and diet during the intervention period. Changes to medications were not recommended unless they were medically indicated. However, blood glucose levels were not measured during the wash-out periods. Therefore, comparisons were made only between groups; changes within groups before and after the intervention were not analyzed. In addition, CGM was applied to only 30 participants because of attachment errors, refusals, and other factors. The core indicators of CGM were analyzed according to the guidelines and interpretation criteria of the Advanced Technologies and Treatments for Diabetes Congress [17].

### 2.7. Statistical Analyses

Continuous outcome variables are presented as means and standard deviations, while categorical variables are presented as frequencies (percentages). Changes before and after the intervention in the two groups were assessed using paired t-tests. For the CGM results, the Student’s *t*-test or Mann–Whitney U test was conducted to assess differences. All analyses were performed using IBM SPSS Statistics version 25.0 (IBM Corp., Armonk, NY, USA), and significance was set at *p*-values of <0.05.

## 3. Results

### 3.1. General Characteristics

Table 1 shows the data on the general characteristics of the subjects (n = 40). The sample comprised of 22 (55.0%) males and 18 (45.0%) females. The participants’ average age was 61.4 ± 10.4 years; 18 (45.0%) of them were older than 65 years, and 22 (55.0%) participants were in the 40–60-year-old range. Most participants were married, but only 1 (2.5%) of them were unmarried, while 7 (17.5%) were bereaved and 3 (7.5%) were divorced. Further, 36 (90.0%) of participants had attained high school-level education or higher, and 4 (10.0%) of them each had an elementary school and middle school education. The average duration of diabetes was 7.1 ± 7.7 years, and approximately 20 (50.0%) of declared treatments involved the use of oral hypoglycemic agents. Additionally, 1 (2.5%) involved insulin and 4 (10.0%) involved the combined use of insulin and oral hypoglycemic agents. Regarding lifestyle management, 10 (25.0%) of the participants adopted a comprehensive approach, whereas 5 (12.5%) followed only dietary therapy. Only 5 (12.5%) of the participants experienced diabetes-related complications. The current smoking and alcohol consumption rates were 2 (5.0%) and 19 (47.5%), respectively. It was found that non-smokers were 28 (70.0%) and those with past smoking experience were 10 (25.0%). Regarding alcohol consumption, non-drinkers were 15 (37.5%), and participants who had consumed alcohol in the past were 6 (15.0%). The proportion of individuals engaging in regular exercise was 28 (70.0%). Based on these results, most participants were considered “diligent” at self-management.

### 3.2. Comparison of Clinical Factors and Nutritional Status Changes

Table 2 shows the results of analyzing the clinical indicators and nutritional status of the subjects. In the intervention group, there was a significant decrease in waist circumference from 86.7 ± 9.3 to 85.8 ± 9.0, and the body fat percentage also showed a significant reduction from 19.0 ± 7.2 to 17.2 ± 6.7. Upon comparing the LDL cholesterol, triglycerides, and average blood glucose levels before and after the intervention in the intervention group, significant decreases were noted. In the control group, there was a slight increase in body weight, systolic blood pressure, diastolic blood pressure, and LDL cholesterol levels. The average overall nutritional score was 63 points. The scores were comparable before and after the intervention in the intervention group, while the average and practice scores improved in the control group.

### 3.3. Changes in Nutrient Intake

The average daily nutrient intake per group, including the meals provided, is shown in Table 3. There were no significant changes in energy, carbohydrate, protein, or fat intake in either group after the intervention. However, in the intervention group, the carbohydrate energy ratio slightly decreased from 56.6 ± 4.2 to 53.6 ± 4.1, and the protein energy ratio increased from 18.7 ± 2.5 to 20.5 ± 3.8, and these changes were statistically significant. Additionally, there was a significant reduction in sugar intake, decreasing from 45.3 ± 11.8 to 40.1 ± 7.1.

### 3.4. Analysis of Blood Glucose Index Changes

**Figure 2:**
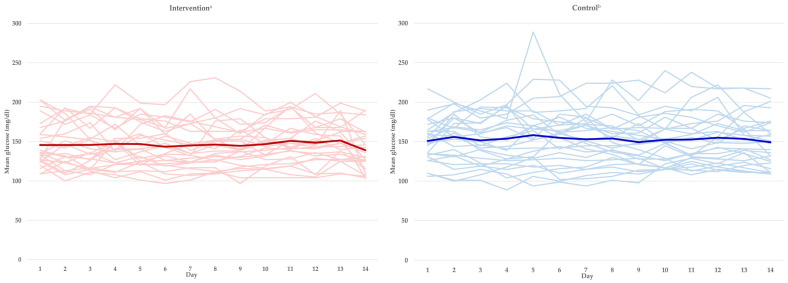
The individual daily glycemic averages and the overall glycemic mean between the two groups. ^a^ The pink line denotes the individual daily glycemic averages over a period of 14 days, and the red line indicates the overall glycemic mean for the same 14-day period. ^b^ The sky blue line denotes the individual daily glycemic averages over a period of 14 days, and the blue line indicates the overall glycemic mean for the same 14-day period.

The CGM values are shown in Table 4 and Figure 2. Figure 2 presents a graph of the daily average blood glucose levels over different periods and the average blood glucose levels by group, based on a standard diet or a diabetic diet, referencing prior studies [18]. The diabetes treatment guidelines refer to three sets of values for effective blood glucose management [19]: percentage of readings and time per day within the target glucose range (70–180 mg/dL), and percentage of time above the target glucose range (180–250 mg/dL), percentage of time in the high glucose range (≥250 mg/dL). During the meal intake period, the intervention group met the requirements defined by all three ranges, and the intervention group showed results exceeding 6.3%. Significant differences between the two groups were analyzed to be significantly lower in the percentage of time above the target glucose range (180–250 mg/dL).

## 4. Discussion

Diabetes is a serious, chronic, and progressive disease accompanied by various complications that increase personal and societal burdens. The ultimate goal of diabetes treatment is to improve health status and quality of life by preventing or delaying the onset of complications [20]. To achieve this goal, various treatment targets, including blood glucose levels, blood pressure, lipid levels, and weight, must be addressed [21]. Dietary management is essential for achieving these goals in patients with diabetes. In this study, we aimed to examine the effectiveness of meal plans designed according to the diabetes management guidelines in supporting the self-management of type 2 diabetes. Most participants in this study were aged ≤65 years. The average age was 61.4 years, and the average duration of diabetes was 7.1 years. The risk of complications increases exponentially with the duration of diabetes. Beyond the first decade, the risks of stroke and death increase 1.1–1.5 times in patients with type 2 diabetes, and those of myocardial infarction and heart failure increase 1.5–2.0 times [22,23]. The complication rate among study participants was relatively low (12.5%). Furthermore, the participants were considered committed to their health management, given their low smoking rate and high regular exercise rate.

Post-intervention, the intervention group showed a significant decrease in waist circumference and body fat percentage. In contrast, the control group experienced significant deterioration in some aspects of its blood lipid status over time. In a randomized controlled trial investigating the effects of a Mediterranean diet on cardiovascular disease in patients with diabetes, the intervention group showed a 31% reduction in the risk of cardiovascular disease compared to the control group [24]. Guidelines for lipid management include reducing the intake of saturated fatty acids, cholesterol, and trans fats while increasing the intake of omega-3 fatty acids and dietary fiber [25]. A high intake of carbohydrates is a potent obesogen (obesity-inducing substance) as it increases the mass of WAT and leads to the most significant weight gain compared to other types of feed induction [26]. Given the carbohydrate composition of the intervention diet, the intervention group likely benefited from the changes to their carbohydrate intake.

The NQ (Nutritional Quotient) used in this study helps assess dietary habits by evaluating the usual dietary intake and specific eating behaviors of adults. This assessment method calculates scores across four critical domains: balance, moderation, practice, and the overall nutritional index. These scores are then methodically classified into one of three distinct categories: a “low” grade, which encompasses scores ranging from 0% to 24.9%; a “medium” grade for scores between 25.0% and 74.9%; and a “high” grade, designated for scores from 75% to 100% [27]. Notably, in a comprehensive study focusing on Korean adults and employing the NQ, the aggregate score for the nutritional index was documented at 53.2 points [28]. Although the participants in this study achieved scores that exceeded this average, thereby positioning them within the “medium” grade spectrum, no noteworthy disparities in score changes were detected within the group subjected to the intervention. Conversely, significant improvements in total and practice scores in the control group suggest that emphasizing the importance of dietary regulation through common nutritional education and counseling to the study participants may have contributed to overall awareness and self-management.

The American Diabetes Association advocates for nutritional therapy aimed at adults diagnosed with diabetes, emphasizing the importance of maintaining a healthy weight through the careful selection and consumption of a diverse range of fundamental nutrients in precise quantities [29]. In contrast, authoritative bodies such as the International Diabetes Federation and the World Health Organization have clarified that there is no groundbreaking dietary therapy specifically for the management of diabetes. Despite this, the established dietary guidelines for those living with diabetes suggest an intake comprising approximately 45–65% of the total caloric intake from carbohydrates that are rich in fiber, 20–35% from fats, and 15–25% from proteins [30]. These guidelines aim to promote balanced nutrition and effective diabetes management. In our study, notably within the intervention group, outcomes such as a decrease in the proportion of total calories derived from carbohydrates, an increase in the proportion of calories obtained from protein, and a significant reduction in overall sugar intake are regarded as beneficial results aligned with these dietary recommendations.

Diabetes management in South Korea is extremely poor. The awareness rate among adults aged ≥30 years is 65.8%, while the treatment rate is 61.4% [3]. However, the control rate among the patients undergoing treatment has been estimated at 24.5%, indicating a very low level of control [30]. Glycemic control targets for adults with type 2 diabetes are glycated hemoglobin levels of <6.5% [3]. For those using CGM devices, it is recommended that the time spent within the target blood glucose range of 70–180 mg/dL should exceed 70%, with less than 4% of the time spent below this range (<70 mg/dL) and less than 1% of the time spent below the level of 54 mg/dL [31]. Our study participants were within the recommended limits at baseline, and the intervention group spent less time above the target glucose range compared to the control group. Similar national studies have shown improved average blood glucose levels and time spent within the target blood glucose range (70–180 mg/dL) when patients with type 2 diabetes were instructed to consume delivered meals. A separate randomized crossover trial conducted over 7 days with high-carbohydrate, low-carbohydrate-high-protein, and low-carbohydrate-high-fat diets showed that most participants had a lower time above the target range when consuming the low-carbohydrate-high-protein diet, which is similar to the results of our study [10]. CGM is recommended for monitoring patients with type 2 diabetes who do not receive insulin therapy. However, to verify this, self-blood glucose monitoring was conducted in parallel, and this improved in the intervention group. This suggests that decreased carbohydrate intake and increased protein intake among the study participants were indirectly associated with a significant reduction in the time above the target range ratio measured by CGM. Blood glucose self-monitoring helps patients understand the impact of their dietary habits, exercise, and diabetes medications on blood glucose control. In addition, it allows to detect hypoglycemia and monitor blood sugar fluctuations in the presence of comorbidities. It can also be useful in cases where the accuracy of glycated hemoglobin values is unclear. Blood glucose monitoring should be individualized. For patients with uncontrolled diabetes who require more aggressive meal management, it is crucial to observe changes in blood glucose levels based on food intake.

The study faces several limitations, including a relatively small sample size of 40 participants, a brief duration of merely 2 weeks, and the inclusion of a washout period that potentially allows for the lingering effects of the dietary intervention to influence the results. Moreover, the variety of meal options was somewhat restricted, with participants having only two of their three daily meals regulated within the study parameters. Despite these constraints, considerable efforts were undertaken to tailor the dietary intake to the specific needs of each participant, coupled with comprehensive education on glucose monitoring techniques aimed at minimizing errors in data collection. The utilization of Continuous Glucose Monitoring (CGM) technology remains a pivotal component in the effective management of diabetes. For future research, it is recommended that studies extend beyond a 3-month period and incorporate larger participant groups to substantiate the findings of the current study. Additionally, these studies should focus on assessing critical health outcomes such as the decrease in levels of glycosylated hemoglobin and enhancements in associated comorbid conditions. This evidence can help determine the role of dietary interventions in diabetes management and outcomes.

## 5. Conclusions

Tailored dietary interventions can help patients with diabetes improve their clinical factors, such as waist circumference, body fat percentage, LDL cholesterol, triglyceride, and average blood glucose levels. From the perspective of continuous glucose monitoring (CGM), eating tailored meals for diabetes patients can reduce the percentage of time when glucose levels are above the target range of 180–250 mg/dL. The meal-type foods for diabetes patients slightly decrease the carbohydrate energy ratio and sugar intake and increase the protein energy ratio. In Korea, different food products for patients with diabetes are being developed based on the diabetes management guidelines, which show positive effects on health indicators, including the control of blood glucose levels. This study provides evidence that supports the use of dietary interventions in the treatment of diabetes, which can help inform product development and patient decision-making.

## Figures and Tables

**Table 1:** General characteristics.

Variables	Subjects (n = 40)
Gender	
Male	22 (55.0%)
Female	18 (45.0%)
Age (yrs)	61.4 ± 10.4
40~64	22 (55.0%)
≥65	18 (45.0%)
Married status	
Married	29 (72.5%)
Bereavement	7 (17.5%)
Divorce	3 (7.5%)
Unmarried	1 (2.5%)
Education level	
Elementary school	2 (5.0%)
Middle school	2 (5.0%)
High school	13 (32.5%)
College	23 (57.5%)
Average duration of DM ^a^ (yrs)	7.1 ± 7.7
Treatment	
Insulin	1 (2.5%)
Oral hypoglycemic agent	20 (50.0%)
Insulin + OHA ^b^	4 (10.0%)
Life habit management	10 (25.0%)
Only diet therapy	5 (12.5%)
Complications	
Cardiovascular	1 (2.5%)
Retinal	1 (2.5%)
Neurovascular	1 (2.5%)
Hypertension	2 (5.0%)
Smoking	
Smoker	2 (5.0%)
Non-smoker	28 (70.0%)
Ex-smoker	10 (25.0%)
Drinking	
Drinker	19 (47.5%)
Non-drinker	15 (37.5%)
Ex- drinker	6 (15.0%)
Exercise	
Regular	28 (70.0%)
Irregular	6 (15.0%)
None	6 (15.0%)

Data were reported as mean ± standard deviation for continuous variable and percentage for categorical variable. ^a^ Diabetes Mellitus. ^b^ Oral Hypoglycemic Agent.

**Table 2:** Clinical factors and nutritional status.

Variables	Intervention (n = 40)			Control (n = 40)		
	Pre	Post	*p*-Value	Pre	Post	*p*-Value
Cardiovascular risk factors						
Weight (kg)	67.5 ± 13.7	67.6 ± 12.8	0.716	67.2 ± 13.3	68.9 ± 13.0	<0.001
Waist circumference (inch)	86.7 ± 9.3	85.8 ± 9.0	0.023	86.7 ± 8.7	86.4 ± 9.6	0.627
Body Fat (%)	19.0 ± 7.2	17.2 ± 6.7	<0.001	18.1 ± 7.1	17.6 ± 6.9	0.323
Systolic Blood Pressure (mmHg)	129.6 ± 13.0	130.4 ± 13.8	0.601	126.7 ± 11.5	134.5 ± 16.2	0.001
Diastolic Blood Pressure (mmHg)	76.0 ± 9.7	76.8 ± 9.3	0.363	75.5 ± 9.8	80.4 ± 10.8	0.001
Total cholesterol (mg/dL)	163.2 ± 33.9	157.6 ± 32.5	0.056	157.0 ± 33.5	163.2 ± 30.8	0.065
LDL cholesterol (mg/dL)	111.8 ± 35.0	102.5 ± 36.4	0.004	108.2 ± 34.3	114.2 ± 33.7	0.034
HDL cholesterol (mg/dL)	53.6 ± 12.5	52.7 ± 14.3	0.459	53.0 ± 13.3	54.9 ± 12.2	0.110
Triglyceride (mg/dL)	190.2 ± 71.2	174.7 ± 68.2	0.001	178.6 ± 80.4	184.6 ± 64.6	0.349
SMBG ^a^ (mg/dL)	178.1 ± 55.7	137.8 ± 22.2	<0.001	183.6 ± 46.2	178.1 ± 55.7	0.572
Nutritional status factors						
Nutritional score	63.9 ± 12.3	64.0 ± 11.8	0.897	62.4 ± 12.9	65.0 ± 11.8	0.026
Balance	52.5 ± 17.6	51.9 ± 16.9	0.745	50.3 ± 17.4	52.0 ± 17.4	0.283
Moderation	71.5 ± 14.6	73.2 ± 13.7	0.271	72.8 ± 15.0	73.9 ± 14.0	0.496
Practice	66.7 ± 17.2	66.1 ± 16.0	0.731	63.6 ± 17.9	68.1 ± 16.2	0.021

Data were reported as mean ± standard deviation for continuous variable. *p*-value was calculated by paired *t*-test for continuous variable as appropriate. ^a^ Self-Monitoring Blood Glucose at 2 h after diet.

**Table 3:** Nutrient intakes.

Variables	Intervention (n = 40)			Control (n = 40)		
	Pre	Post	*p*-Value	Pre	Post	*p*-Value
Energy (kcal)	1911.2 ± 328.2	1867.8 ± 247.0	0.506	1812.6 ± 382.9	1837.8 ± 310.6	0.658
Carbohydrate (g)	262.1 ± 54.0	258.6 ± 48.6	0.721	269.9 ± 45.8	266.5 ± 48.1	0.670
Sugar (g)	45.3 ± 11.8	40.1 ± 7.1	0.005	44.7 ± 14.8	41.7 ± 13.7	0.127
Protein (g)	89.5 ± 17.2	92.1 ± 16.3	0.459	75.3 ± 25.7	74.6 ± 20.4	0.857
Fat (g)	56.0 ± 14.9	56.3 ± 11.5	0.933	44.5 ± 18.1	47.5 ± 18.11	0.394
Carbohydrate energy ratio (%)	56.6 ± 4.2	53.6 ± 4.1	0.007	61.0 ± 9.0	59.0 ± 8.0	0.221
Protein-energy ratio (%)	18.7 ± 2.5	20.5 ± 3.8	0.047	16.0 ± 3.0	16.0 ± 3.0	0.587
Fat energy ratio (%)	24.7 ± 3.4	25.4 ± 2.2	0.312	22.0 ± 6.0	23.0 ± 7.0	0.365

Data were reported as mean ± standard deviation for continuous variable. *p*-value was calculated by paired *t*-test for continuous variable as appropriate.

**Table 4:** Blood continuous glucose monitoring status.

Variables	Target [17]	Intervention (n = 30)	Control (n = 30)	*p*-Value
Mean glucose (mg/dL)	-	146.1 ± 28.9	157.5 ± 28.0	0.127
Target range (70~180 mg/dL, %)	>70%	80.2 ± 18.5	75.4 ± 19.5	0.336
Above range (180~250 mg/dL, %)	<25%	12.1 ± 9.2	18.0 ± 12.8	0.045
Above range (≥250 mg/dL, %)	<5%	4.3 ± 7.2	6.3 ± 8.7	0.342
Mean glucose management indicator (GMI, %)	-	6.7 ± 0.6	7.0 ± 0.7	0.100

Data were reported as mean ± standard deviation for continuous variable. *p*-value was calculated by Student’s *t*-test or Mann–Whitney U test for continuous variable as appropriate.

## Data Availability

Data are available upon reasonable request. The individual de-identified participant data will be shared with the corresponding author upon reasonable request. The data is not shared publicly due to privacy reasons.

## Supplementary Materials

The following supporting information can be downloaded at: https://www.mdpi.com/article/10.3390/nu16081190/s1, Table S1: Information on meal-type food with diabetes patients.
