# Supplementary material for: Tailored Meal-Type Food Provision for Diabetes Patients Can Improve Routine Blood Glucose Management in Patients with Type 2 Diabetes: A Crossover Study
Source: Nutrients. 2024 Apr 17;16(8):1190. doi: 10.3390/nu16081190 (PMC11055165; doi:10.3390/nu16081190)
Supplement: Supplementary file 1 [file nutrients-16-01190-s001.zip › nutrients-2931349-supplementary.pdf]

Table S1: Information of meal-type food with diabetes patients

No	Meal name	Weight (g)	Energy (kcal)	Carbohydrate (g)	Protein (g)	Fat (g)	Saturated Fat (g)	Trans Fat (g)	Sugar (g)	Sodium (mg)	Cholesterol (mg)
1	Stir-fried spicy pork & rice made of oat and konjac	412	635	73	36	22	4.5	0	9	910	75
2	Dak-galbi (spicy stir-fried chicken) & rice made of black rice and konjac	396	630	84	29	20	3.1	0	10	910	110
3	Tteokgalbi(Korean meat patties) & rice made of brown rice and konjac	387	630	86	22	22	6	0	11	1140	55
4	Jjajangmyeon (black bean noodles) made with tofu noodles	445	625	53	38	29	5.5	0	11	1230	30
5	Grilled chicken breast & rice made of barley and konjac	391	605	78	31	19	5.1	0	9	560	105
6	Pork bulgogi & rice made of black rice and konjac	400	535	67	28	17	4.6	0	8	840	75
7	Soybean paste sauce & barley rice	442	525	69	28	15	4.2	0	8	1040	40
8	Spicy chicken & rice made of brown rice and konjac	346	525	78	29	11	2.3	0	6	990	95
9	Tteokbokki& grilled chicken breast	352	515	72	28	13	3.3	0	9	830	100
10	Jjimdak(Korean braised chicken with vegetables) & rice made of oat and konjac	404	510	70	35	10	3.1	0	8	850	95
